# Toward a Defined Role for Occupational Therapy in Foster Care Transition Programming

**DOI:** 10.15453/2168-6408.1726

**Published:** 2020

**Authors:** Amy Armstrong-Heimsoth, Molly Hahn-Floyd, Heather J. Williamson, Catherine Lockmiller

**Affiliations:** Northern Arizona University – USA; Northern Arizona University – USA; Northern Arizona University – USA; Northern Arizona University – USA

**Keywords:** aging-out, transition, foster care, participation

## Abstract

Youth who age out of the foster care system and transition to adulthood face challenges that are exacerbated by a history of trauma, severed relationships, and instability of living and educational placements. A review of the literature demonstrates poor outcomes overall for this population. Occupational therapists are positioned to meet the needs that arise during this time; however, a review of emerging roles for occupational therapists is necessary to describe how occupational therapists can best fulfill gaps in current programming. Through a review of the literature and a preliminary mixed-methods study, this paper establishes a direction for the inclusion of occupational therapy for youth aging out of foster care using the Person Environment Occupation Performance (PEOP) model as a structure. Federal, state, and local organizations provide resources to assist transitioning foster youth. However, there is a lack of collaborative, individualized, and evidence-based approaches reporting good outcomes. Specific occupational therapy interventions are suggested to delineate our role with this high-risk population during transition to independent living: both novel interventions and additions to current evidence-based programming.

Every year approximately 24,000 foster care youth age out of the foster care system, which generally occurs at or around 18 years of age but can occur at up to 21 years of age in some states (U.S. Department of Health & Human Services Children’s Bureau, 2019). The child welfare system allows 90 days for determination of a case plan to transition out of the system. During this period, a team is constructed and options for support are presented. The individual must then decide to continue in the system via supplemental supports or opt out of support.

In either case, youth must quickly adapt to independent living constraints involving housing, health insurance, employment, transportation, and education. There is a valid reason for youth to moderate this process, as remaining in care past the age of 21 has been associated with higher earnings and educational achievement. Unfortunately, reports indicate that the average age of transition in nearly every state is at least 1 year lower than the maximum allowable age (Fryar et al., 2017).

Transition programs currently available for youth are categorized by the American Youth Policy Forum (AYPF) in three primary areas of transition programming: sustainable social capital, permanency supports, and postsecondary access and success (Russ & Fryar, 2014). Sustainable social capital transition programs include mentorship, self-advocacy, life skills, and well-being programs. Permanency supports transition programs include housing, health care, transportation, employment, and financial supports. Postsecondary access and success transition programs involve vocational training, interviewing skills, college assistance, and tutoring (Russ & Fryar, 2014). Without these programming initiatives, youth who opt to receive long-term transition support are left without the tools to impact success. At the same time, young people are conditioned to mistrust institutions and opt out of support (Braciszeweski et al., 2018; Courtney et al., 2005; Gomez et al., 2015). This results in a vicious cycle wherein lack of support leads to opting out, which negatively affects outcomes and hampers justification for funding and program development.

Even when programming is used, transitional outcomes are bleak. Twenty percent will become homeless immediately after transition, 50% will be unemployed at 24 years of age, 50% will go on to develop a substance dependence, 25% will be diagnosed with PTSD, 70% of women will become pregnant before 21 years of age, only 46% will earn a high school diploma, and less than 3% will obtain a bachelor’s degree (Courtney et al., 2007). Behavioral health service dropout rates for youth after transition is estimated at 60% (Havlicek et al., 2013). Taken together, these data express a complex interplay between social systems that work together to disempower foster youth. Structural change is necessary, but individuals can produce sustainable change at the individual and local levels. As we will show in the following sections, occupational therapists can bring our unique lens in all three transition areas (sustainable social capital, permanency support, and postsecondary access and success), thereby maximizing the capacity of high risk youth.

## Literature Review and Preliminary Study

This opinion piece is informed by a review of the literature and a preliminary study. The review was organized with help from a health sciences librarian specializing in rehabilitative health professions and followed from the research question: “What are best practices in occupational therapy for assisting young adults transitioning out of the foster care system?” The study team completed a review of existing legislation, practice guidelines, and the research literature regarding transitions for foster youth populations. Search results indicated a nationwide, structural lack of support for foster youth, a lack of evidence-based programming, and a widespread need for the specialized services that occupational therapists bring to the table.

Permanency support programs are integral to lifelong success. However, the literature suggests that they rarely balance structural inequities experienced by foster youth. For instance, one program, Youth Villages, operates across seven states and provides comprehensive, transitional living support (Skemer & Valentine, 2016). The program assists with stable housing, employment, life-skill building, and health management. A follow-up survey did not indicate that Youth Village’s program had an impact on education, social support, or criminal involvement (Skemer & Valentine, 2016). However, it did lead to increases in earnings and employment as well as reduced homelessness 1 year following (Skemer & Valentine, 2016). In addition, 63% of participants in the Midwest Study reported at least one employment or vocational support while in care (Dworsky & Havlicek, 2010). After leaving care, those numbers decreased to 43% (Dworsky & Havlicek, 2010). Eighty-four percent of participants in the Northwest Foster Care Alumni Study reported having access to employment training or job location services (Center for Mental Health Services and Center for Substance Abuse Treatment, 2013). Less than 20% of foster care alumni in Utah reported receiving employment support services within 3 years of leaving care (Dworsky & Havlicek, 2010). These results suggest that sustained permanency is exceedingly difficult to achieve, even when permanency support is available. Therefore, it is imperative that we examine factors that contribute to poor outcomes.

Previous studies have reported barriers to participating in transition services, including lack of trust, learned helplessness, and displacement pre and post discharge (Braciszewski et al., 2018; Courtney et al., 2005; Gomez et al., 2015). However, research indicates that empowering youth throughout the transition process, providing opportunities to develop independent living skills, fostering positive relationships with a mentor, and increasing placement permanency affect the likelihood of post discharged foster youth pursuing transitional services (Gomez et al., 2015). As a result, occupational therapists have a responsibility to develop programs that build resilience in youth while collaborating more closely with placement services.

Predictive factors for foster youth pursuing educational, vocational, and mental health services are nearly identical. Foster youth who remain in care for the first 6 months post discharge receive financial services, feel close to at least one person, are satisfied with their experience in foster care, and are more likely to pursue educational and vocational services (Courtney et al., 2005). Further, foster youth with educational or vocational aspirations are more likely to pursue vocational services, which has implications for occupational therapy interventions relating to organization, resilience, and change management (Courtney et al., 2005). Positive predictive factors for pursuing mental health services include staying in care for the first 6 months post discharge and increasing accessibility to telehealth and online health care services (Braciszewski et al., 2018; Courtney et al., 2005). Barriers include perceived cost of care, no health insurance, and poor provider-client bond, which specifically may be affected by lack of delivery, coordination or continuity of care, and housing instability (Braciszewski et al., 2018; Courtney et al., 2005).

In addition to the literature review, the authors conducted and published a preliminary study examining youth’s perceptions of transition programming, which also bears implications for this opinion piece. Sixteen youth who had aged out in the previous 2 years were interviewed as a part of the mixed-methods study. Eight participants had transition services, eight either delayed receiving transition supports or did not receive any support. Important themes emerged suggesting a need for transition programming to begin earlier and to include modalities designed to increase self-determination. All youth reported a need for person-centered services. Desired services included assistance with mental health resources, housing, education, transportation, food, finances, social skill building, and instrumental activities of daily living (Armstrong-Heimsoth et al., 2020). This study, along with the literature review, indicates the need for robust, evidence-based service delivery.

## Occupational Therapy Opportunities for Foster Care Youth

Placement into the foster care system leads to disruption, loss of a sense of permanency, and long-term trauma. Each new placement in care further compounds these disparities and restricts self-determination (Lynch et al., 2017; Stott & Gustavsson, 2010). More placements compound adverse experiences and necessitate the need for formal supports after age 18 (Stott, 2009). With less than 40% of states able to sustain two or fewer placements while in care, occupational therapists can play a critical role in assisting with mitigating impacts and successfully transitioning foster youth into adulthood. All transition areas identified by the AYPF are encompassed in one of occupational therapy’s main areas of focus as defined by the *Occupational Therapy Practice Framework* (OTPF-3) (American Occupational Therapy Association [AOTA], 2014).

Occupational therapists are well established in providing transition services for other high-risk populations, such as adolescents with disabilities under the Individuals with Disabilities Education Act. In this capacity, an occupational therapist “builds students’ abilities to transition to new settings, establish necessary roles and routines, and participate in activities that will support independence in home, work, and community environments through adulthood” (Podvey & Myers, 2018, p. 4). There is a potential for youth in foster care to interface with occupational therapists in the school setting through the individualized education plan (IEP) process. However, in this capacity occupational therapy services are limited to supporting the child’s success in school and are not focused on their skills necessary outside of school for successful community living. Therefore, the authors propose that occupational therapists be used in a community supports capacity rather than an embedded educational role.

The expertise among occupational therapists in social capital supports is also well established. Self-advocacy is a defined intervention in the OTPF-3, and well-being is an overarching theme used to define occupational therapy practice (AOTA, 2014). Further, the AOTA *Vision 2025* states, “As an inclusive profession, occupational therapy maximizes health, well-being, and quality of life for all people, populations, and communities through effective solutions that facilitate participation in everyday living” (AOTA, 2019, n. p.). This vision speaks directly to current needs for youth aging out of foster care and calls on occupational therapists to effect change at all service delivery levels (AOTA, 2014).

Because occupational therapists are able to use a multi-level and ecological approach, they are better prepared to assist youth in transition. Occupational therapists are skilled at assessing environments to ensure performance or provide necessary supports. In addition, occupational therapists’ attention to social participation and the context of the social environment is unique to current professions engaged in the transition process. This systems-level approach to transitional support is best understood through the Person Environment Occupation Performance (PEOP) model.

The PEOP model describes relationships among person factors, environmental factors, and occupations (Baum et al., 2015). Occupational performance is central to overall well-being and contributes to an individual’s identity, roles, tasks, and actions. The emphasis on performance is central and unique to the PEOP model and is defined as “the doing of meaningful activities, tasks, and roles through complex interactions between the person and environment” (Baum et al., 2015, p. 52). The use of this lens is crucial in creating the individualized person-centered programming youth are seeking.

The PEOP model interventions focus on dynamic relationships and how they affect occupational performance. This fills a gap in current transition programming. While there are multiple foster care transition programs and services across the United States, we can find no evidence of performance or participation reported as outcomes. In addition, none can be found that examine goodness of fit between youth and the transition supports as a contributory factor to successful participation.

Tables 1-3 summarize proposed PEOP model intervention strategies for the persons, groups, and population levels based on the intervention categories, including: establish, adapt, alter, prevent, create, educate, and advocate (Baum et al., 2015). Outlined are services that occupational therapists could create (are not currently provided) and existing programming to which occupational therapy could contribute. Persons-level services include individualized assessments, interventions, and environmental modifications in each of the AYPF defined areas of transition need. Because of a history of trauma, severed relationships, and instability as a result of multiple placements, specific client factors need to be addressed at the persons-level for improved performance (Fryar et al., 2017). Emotional regulation, social skills, independent living skills assessments, interventions, and environmental modifications are strongly recommended to improve occupational performance and outcomes (Fryar et al., 2017). Performance skills, including social interaction and executive function skills, that address goal setting and planning would improve future performance in independence in sustainable social capital and postsecondary success. The Urban Institute report calls for employment programs to pay particular attention to specific individual challenges and barriers to employment (Edelstein & Lowenstein, 2014). This includes soft skills of employability, such as conflict resolution, self-advocacy, emotional regulation, and interpersonal skills for collaborative work environments (Edelstein & Lowenstein, 2014). Instrumental activities of daily living and life skills training are necessary to assess and increase skill for successful independent living. Finally, addressing activity patterns and establishing roles and routines would improve performance in all three areas of transition need, in particular, instrumental activities of daily living.

Groups-level service would require consultation with professionals who regularly interact with pretransition youth. Provision of training with case managers, group home staff, and transition team members could develop screening resources for specific client factors or performance skills and toolkits for organizations to improve performance skills, such as social and communal and health wellness promotion. Table 2 proposes specific screening resources and toolkits not currently provided to professionals working with pretransition foster youth.

At populations-level, occupational therapists consult with federal and state systems in a critical role that involves recommending system-wide screenings, resources and toolkits, environmental modifications, and implementing and disseminating evidence of successful programs. In addition, resources or trainings are needed for foster youth to lessen the impact of a system that may inhibit development of motivation, self-efficacy, and decision-making skills because of a lack of opportunities to be independent and take risks. Occupational therapists may also recommend systems level measurement tools that focus on performance and outcomes of current transition programs.

### Conclusion

Aging out of foster care has well documented challenges and is compounded by a range of adversities. This opinion piece supports previous work promoting the role of occupational therapy in foster care and further proposes intervention strategies supported by a literature review and a preliminary study (Paul-Ward & Lambdin-Pattavina, 2016). Use of the PEOP model can assist in organizing future research endeavors related to outcomes and implementation. It provides fidelity and, therefore, increased understanding of occupational therapy’s unique perspective to maximize our contributions in an emerging practice area.

Occupational therapy can assist former foster youth in improved occupation, participation, and health in adulthood. Expanding our role in foster care requires nontraditional settings, advocacy work, and pursuit of grant funding. Finally, it will be important to tie the role of occupational therapy to larger policy goals, including the AYPF transition outcomes of sustainable social capital, permanency supports, and postsecondary opportunities. All three of these policy goals align with our scope of practice and our strengths in intervening from an occupation based ecological model. Occupational therapists are an existing evidence-based resource in all communities that can provide supportive environments to address three AYPF transition outcomes for foster system youth.

## Figures and Tables

**Table 1:** Occupational Therapy Strategies to Fill Gaps in Transition Programming - Persons Level

Occupational Therapy’s Role	PEOP Intervention Categories	Intervention Strategies
Individualized assessment	Create Establish	**Audience:** Individual youth, transition team **Topics:** Client factors Emotional regulation Executive function Performance patterns Roles Routines Self-advocacy Interpersonal skills Leisure exploration Interest inventories Health management and maintenance Health literacy
Individualized intervention	Create Establish	**Audience:** Transition team and individual, group home and individual **Topics:** Client factors Emotional regulation Executive function Performance patterns Roles Routines Life skills Social participation Self-advocacy Leisure exploration Interest inventories Health management and maintenance Health literacy Goal setting Individualized resource list
Individualized environmental modifications	Create Establish Advocate Educate	**Audience:** Transition team and individual, group home and individual Maximizing client and environment factors to increase goodness of fit and maximize performance

**Table 2:** Occupational Therapy Strategies to Fill Gaps in Transition Programming - Groups Level

Occupational Therapy’s Role	PEOP Intervention Categories	Intervention Strategies
Provision of in-services	Advocate Educate	**Audience**: Clinics, welfare agencies, congregate care staff and supervisors, transition teams, schools **Topics:** Maximizing occupational performance
Training or education programming	Create Establish	**Audience:** Clinics, welfare agencies, congregate care staff and supervisors, transition teams, schools **Topics:** Client factors Performance patterns Life skills Social participation Self-advocacy Interpersonal skills Health management and maintenance Health literacy
Develop screening resources and toolkits for specific county/city/district	Create Establish	**Topics:** Life skills Social participation Healthy relationship skills Employment interests and pursuits Interview skills Vocational training Conflict resolution Self-advocacy Interpersonal skills Financial management Budgeting Leisure exploration Interest inventories Health management and maintenance Wellness Health literacy Self-advocacy
Broaden scope of occupational opportunity	Promote	Develop resources based on occupational opportunity categories

**Table 3:** Occupational Therapy Strategies to Fill Gaps in Transition Programming - Populations Level

Occupational Therapy’s Role	PEOP Intervention Strategies	Primary Strategy
Consult with federal and/or state system	Advocate Educate Establish Promote Create	Client centered strategies and resources for transition process Establish a how-to guide or education program to increase resiliency Development of activities to embed in transition program to promote wellness and well-being, health promotion, and management
Develop screening resources/toolkits	Create Educate Establish Advocate	Occupational engagement toolkit Goodness of fit (task and environment fit) assessments/checklists Self-advocacy manuals (how to teach) Goal setting worksheets/toolkits Mental health toolkit- health management resource
Develop environmental modifications Broaden scope of occupational opportunity	Create Establish Promote	Resource/strategies for adapting and grading tasks to fit environment Develop resources based on occupational opportunity categories
Implement and disseminate evidence of successful programs	Educate Modify Establish	Data tracking models Measurement models for all assessments/checklists/resources

## References

[R1] American Occupational Therapy Association. (2014). Occupational therapy practice framework: Domain and process (3rd ed.). American Journal of Occupational Therapy, 68(Suppl. 1), S1–S48. 10.5014/ajot.2014.682006 12458855

[R2] American Occupational Therapy Association. (2019). Vision 2025. https://www.aota.org/AboutAOTA/vision-2025.aspx

[R3] Armstrong-Heimsoth A, Hahn-Floyd M, Williamson HJ, Kurka JM, Yoo W, & Rodríguez De Jesús SA (2020). Former foster system youth: Perspectives on transitional supports and programs. The Journal of Behavioral Health Services & Research. 10.1007/s11414-020-09693-6 PMC7518090 32095998

[R4] Baum C, Christiansen C, & Bass J (2015). Person-Environment-Occupational Performance (PEOP) Model In Christiansen CH, Baum CM, & Bass JD (Eds.), Occupational therapy: Performance, participation, well-being (4th ed., pp. 49–56). Slack, Incorporated.

[R5] Braciszewski JM, Tzilos Wernette GK, Moore RS, Tran TB, Bock BC, Stout RL, Chamberlain P, & Vose-O’Neal A (2018). Developing a tailored substance use intervention for youth exiting foster care. Child Abuse & Neglect, 77, 211–221. 10.1016/j.chiabu.2018.01.013 29367098 PMC5857233

[R6] Center for Mental Health Services and Center for Substance Abuse Treatment. (2013). Diagnoses and health care utilization of children who are in foster care and covered by Medicaid ([SMA] 13-4804; pp. 1–40). Center for Mental Health Services and Center for Substance Abuse Treatment, Substance Abuse and Mental Health Services Administration https://store.samhsa.gov/product/Diagnoses-and-Health-Care-Utilization-of-Children-Who-Are-in-Foster-Care-and-Covered-by-Medicaid/SMA13-4804

[R7] Courtney M, Dworsky A, & Pollack H (2007). When should the state cease parenting? Evidence from the Midwest study. (Issue Brief No. 115; pp. 1–10). Chapin Hall Center for Children at The University of Chicago https://www.chapinhall.org/research/when-should-the-state-cease-parenting/

[R8] Courtney M, Dworsky A, Ruth G, Keller T, Havlicek J, & Bost N (2005). Midwest evaluation of the adult functioning of former foster youth: Outcomes at age 19 (Midwest Evaluation of the Adult Functioning of Former Foster Youth, pp. 1–77) [Working Paper], Chapin Hall at the University of Chicago. https://www.chapinhall.org/research/at-age-19-youth-transitioning-out-of-foster-care-face-multiple-challenges/

[R9] Dworsky A, & Havlicek J (2010). Employment needs of foster youth in Illinois: Findings from the Midwest study (pp. 1–46). Chapin Hall at the University of Chicago http://citeseerx.ist.psu.edu/viewdoc/download?doi=10.1.1.711.6124&rep=rep1&type=pdf

[R10] Edelstein S, & Lowenstein C (2014). Supporting youth transitioning out of foster care, issue brief 3: Employment programs(OPRE Report #2014-70; Employment Programs, pp. 1–16). Office of Planning, Research and Evaluation, Administration for Children and Families, US Department of Health and Human Services https://files.eric.ed.gov/fulltext/ED559329.pdf

[R11] Fryar G, Jordan E, & Devooght K (2017). Supporting young people transitioning from foster care: findings from a national survey (pp. 1–52) [Executive Summary]. Child trends https://www.childtrends.org/publications/supporting-young-people-transitioning-foster-care-findings-national-survey

[R12] Gomez RJ, Ryan TN, Norton CL, Jones C, & Galán-Cisneros P (2015). Perceptions of learned helplessness among emerging adults aging out of foster care. Child and Adolescent Social Work Journal, 32(6), 507–516. 10.1007/s10560-015-0389-1

[R13] Havlicek J, Garcia A, & Smith DC (2013). Mental health and substance use disorders among foster youth transitioning to adulthood: Past research and future directions. Children and Youth Services Review, 35(1), 194–203. 10.1016/j.childyouth.2012.10.003 23766549 PMC3677527

[R14] Lynch A, Ashcraft R, Paul-Ward A, Tekell L, Salamat A, & Schefkind S (2017). Occupational therapy’s role in mental health promotion, prevention, & intervention with children & youth: Foster care (School Mental Health, pp. 1–3) [Information sheet]. American Occupational Therapy Association, https://www.aota.org/~/media/Corporate/Files/Practice/Children/SchoolMHToolkit/Foster-Care-Info-Sheet-20170320.pdf

[R15] Paul-Ward A, & Lambdin-Pattavina CA (2016). New roles for occupational therapy to promote independence among youth aging out of foster care. The American Journal of Occupational Therapy, 70(3), 7003360010p1–5. 10.5014/ajot.2016.017426 27089300

[R16] Podvey M, & Myers C (2018). Transitions for children and youth: How occupational therapy can help (pp. 1–4). American Occupational Therapy Association https://www.aota.org/~/media/Corporate/Files/AboutOT/Professionals/WhatIsOT/CY/Fact-Sheets/Transitions.pdf

[R17] Russ E, & Fryar G (2014). Creating access to opportunities for youth in transition from foster care: An AYPF policy brief (pp. 1–38) [Policy Brief]. American Youth Policy Forum https://www.aypf.org/resource/creating-access-to-opportunities-for-youth-in-transition-from-foster-care-2/

[R18] Skemer M, & Valentine E (2016). Striving for independence: Two-year impact findings from the Youth Villages Transitional Living Evaluation (pp. 1–80). MDRC https://www.mdrc.org/sites/default/files/Youth%20Villages_2016_FR.pdf

[R19] Stott TC (2009). The well-being and risk behaviors of young adults from foster care. (Publication No. 3361295) [Doctoral Dissertation, Arizona State University]. ProQuest Dissertation Publishing.

[R20] Stott T, & Gustavsson N (2010). Balancing permanency and stability for youth in foster care. Children and Youth Services Review, 32(4), 619–625. 10.1016/j.childyouth.2009.12.009

[R21] U.S. Department of Health and Human Services, Children’s Bureau. (2019). The afcars report: Preliminary fy 2018 estimates as of august 22, 2019 (No. 26; The AFCARS Report, pp. 1–6). U.S. Department of Health and Human Services https://www.acf.hhs.gov/sites/default/files/cb/afcarsreport26.pdf

